# Analysis of Content, Social Networks, and Sentiment of Front-of-Pack Nutrition Labeling in the European Union on Twitter

**DOI:** 10.3389/fnut.2022.846730

**Published:** 2022-04-25

**Authors:** Anggi Septia Irawan, Balqees Shahin, Diana Wangeshi Njuguna, Noel Johny Nellamkuzhi, Bùi Quốc Thiện, Nour Mahrouseh, Orsolya Varga

**Affiliations:** ^1^Department of Public Health and Epidemiology, Faculty of Medicine, University of Debrecen, Debrecen, Hungary; ^2^Doctoral School of Health Sciences, University of Debrecen, Debrecen, Hungary; ^3^Faculty of Medicine, University of Debrecen, Debrecen, Hungary; ^4^Eötvös Loránd Research Network, Budapest, Hungary

**Keywords:** front-of-pack nutrition labeling, European Union, public understanding, European Commission (EC), obesity

## Abstract

In recent years, concerted political efforts have been made at the national and European Union (EU) level to promote the consumption of healthy foods. The European Commission (EC) expressed the need for a harmonized and mandatory front-of-pack nutrition labeling (FOPL) system at the EU level. The EC will adopt the proposal by the end of 2022. Our research work aims to understand the public discourse on FOPL in the EU *via* Twitter, by analyzing tweet content, sentiment, and mapping network characteristics. Tweet search and data collection were performed using the Twitter application programming interface (API), with no time or language restrictions. The content was coded with the QRS Nvivo software package and analyzed thematically. Automatic sentiment analysis was performed with QSR Nvivo, and network analysis was performed with Gephi 0.9.2. A total of 4,073 tweets were posted, mostly from the UK, Spain, and France. Themes that have emerged from the discussion on Twitter include the types of food labeling, food industry, healthy vs. unhealthy foods in the context of food labeling, EU regulation, political conflicts, and science and education. Nutri-Score dominated the discussion on Twitter. General topics were perceived negatively by Twitter users with more positive sentiments toward the food industry, while negative sentiments were observed toward the discourse of political conflicts. The network analysis showed that a centralized communication was hardly existed between countries. Our results reveal that the discussion of FOPL on Twitter is limited to a very limited group of people, and it seems necessary to inform a wide range of consumers about existing and upcoming FOPL schemes. Educational programs should empower consumers to understand what a healthy diet is and how it relates to FOPL, regardless of the existing labeling system.

## Introduction

Since the beginning of the 21st century, marked by a global obesity epidemic and the widespread consumption of ultra-processed foods (1), front-of-pack nutrition labeling (FOPL) initiatives have been growing in both the public and private sectors (2). First proposed by the World Health Organization (WHO) in 2004, FOPL, as a policy measure, was to be applied to improve the nutrition and health of the population (3). The goal of FOPL is usually to provide consumers with additional information to make healthier food choices. It also encourages the industrial sector to make healthier products (4). Despite the recommendations, the use of FOPL is voluntary in most countries, and currently the Codex Alimentarius, which is a collection of standards, guidelines, and codes of practice to be followed by countries around the world, has no explicit requirements for national mandatory FOPL (5).

Currently, all prepackaged foods on the European Union (EU) market must bear a nutrition declaration, in accordance with the requirements set out in Articles 30–34 of the Food Information to Consumers (FIC) Regulation (6). According to Article 32–33, the nutrition declaration shall include at least the energy value and the amounts of fat, saturated fats, carbohydrates, sugars, proteins, and salt per 100 g or 100 ml and shall be presented in a tabular form, where possible with number alignment. However, Article 35 of the FIC allows for additional voluntary repetition of the energy value and/or the amount of nutrients in other expressions and/or in the form of graphics or symbols (e.g., suitability of a food for vegans) to be placed in the front of the food package. Such FOPL should not be misleading, ambiguous, or confusing to consumers, but should be based on relevant scientific data.

In the EU market, the public sector has adopted six forms of FOPL: the Keyhole logo (Denmark Lithuania, and Sweden), Nutri-Score (Belgium, France, Germany, Luxembourg, the Netherlands, and Spain), the heart symbol in Finland, the “Little Heart” logo in Slovenia, the “Healthy Lifestyle” logo in Croatia, and the traffic light scheme (the UK and Ireland). Italy has recently developed a system called NutrInform Battery (7, 8). In May 2020, the European Commission (EC) adopted a report to assess the impacts of FOPL schemes and concluded the need for a harmonized and mandatory FOPL system at the EU level (9). The EC report expressed that the use of color-coding, with or without a graded indicator, seems to be most promising for improving the nutritional quality of food choices, but a single best one has not yet been selected (7, 9). The EC has announced a proposal to harmonize mandatory FOPL in the context of the EU's Farm to Fork strategy (draft action plan), which aims to make food systems fair, healthy, and environmentally sustainable, enabling consumers to stay informed, healthy, and to make sustainable food choices. EC will adopt the proposal by the end of 2022 (10).

Although FOPL policies are considered effective in helping consumers make healthier food choices, Kanter et al. (4) highlighted significant gaps in research addressing FOPL. Interpretive labels are more likely to influence consumer understanding and behavior than reductive schemes alone, less evidence is available on the dynamics of the effectiveness or superiority of different types of interpretive FOPL (11). A study conducted in Uruguay reported that Health Star Rating was worse than Nutri-Score while nutritional warnings and warning labels performed the best. The effect of FOPL schemes for both Nutri-Score and warning labels was dependent on the degree of healthiness of the food, but the effect on consumer behavior for unhealthy product categories was more pronounced for the warning labeling scheme (12). Another study found that product type was the most important factor influencing choice (13). A randomized clinical trial conducted in Bulgaria assessed the objective understanding of five FOPL systems (reference intakes, multiple traffic lights, warning labels, Nutri-Score, and Health Star Rating) in a cohort of 1,010 Bulgarian adults and found that the Nutri-Score was the most effective model to facilitate an understanding of the nutritional quality of foods (14). Similar results were reported in a study from Portugal (15).

Additionally, the link between the effectiveness of FOPL and different socioeconomic status and health literacy levels is still hardly determined, so it is difficult to assess their impact on health inequalities (16). A recent study focused on consumers' perception of the NutrInform Battery, and compared it with Nutri-Score, in seven EU countries (France, Germany, Greece, Italy, Portugal, Romania, and Spain); the findings suggested that the NutrInform Battery can help consumers understand the information in a relevant way, it also performed the best across countries, and the impact of sociocultural differences was limited (17). Furthermore, there has been limited literature on attention-grabbing label characteristics, notably, the required size of labels, the color of labels, or the placement of labels on food packaging (18). To our knowledge, there is no consistent evidence of the absolute “superiority” of one label scheme over others as a prerequisite to changing consumers' dietary habits or, beyond reasonable doubt, reducing overweight and obesity (17).

Because very few researchers examine FOPL in real-world settings (16), a very few studies conducted in online environments are considered really valuable (19). Online studies are easier to conduct and adopt to the local context. These features are crucial as countries that have adopted FOPL systems agree that it is important to test different FOPL formats in-country to ensure that they are appropriate for the country context (20, 21). Social media is an emerging source of knowledge that can influence health outcomes and public understanding (22, 23). WHO launched the global eHealth strategy to encourage the creation, development, and evaluation of health interventions using social media (24, 25). Of all the social network platforms, Twitter was the most appropriate in our study because this network is traditionally used to share ideas, real-time information, and breaking news. Because Twitter is used for professional networking, major institutions, companies, industries, and organizations have Twitter accounts to disseminate information, discuss legislation, or strategically market their services (26). Twitter, as a research data source, has experienced a surge in popularity due to practical reasons, including the ease of use of the platform. Tweets appear in Google search, and a strong hashtag culture simplifies sorting and data collection. Another benefit is the scientific research access offered by Twitter, which allows data collection from Twitter's public real-time data and public historical data and provides additional features to facilitate more accurate data collection. By limiting data collection to selected social media platforms, an analysis will be more transparent because each social media platform has its own analytical tools, which cannot be reliably aggregated (27).

Currently, Twitter is used to facilitate and follow public discussions, evaluate population attitudes towards different measures, network with experts, share opinions, or engage in scientific debates (28–30); it is also used to estimate the impact of promotional information on people's understanding (31). Twitter can also have an influence on health-related issues (32, 33). Available studies indicate that discourse analysis of Twitter data can provide useful information on rapidly changing public sentiments, public attitudes, and concerns (30, 34, 35).

The purpose of this research is to evaluate the public discourse on FOPL in the EU *via* Twitter by analyzing tweet content, sentiment, and mapping network characteristics.

## Materials and Methods

### Study Setting

Twitter's application programming interface (API) was used to search and collect tweet data from November 2021 to December 2021. The search term was Nutriscore OR Keyhole Nutrition labeling OR Nutrition labeling OR front-of-package labeling OR Fop labeling OR Food labeling OR heart symbol Nutrition labeling OR Nutrition labeling traffic light OR Nutrition labeling healthy choices tick OR Healthy living guarantee mark OR Zivjeti zdravo OR Nutrition Labeling Battery. Both public tweets and retweets were incorporated into the data set and included tweets posted in EU countries and the UK as the UK was part of the EU during the public consultation and implementation of FIC. Neither time nor language filter was applied.

### Data Collection

Data were sourced from Twitter by conducting a full archive search endpoint available on the track of academic research products using the Twitter API V2.0. This platform provides a set of API keys and Auth 2.0 bearer token to tweets searched from Twitter API v2.0 Postman version 9.3.1 collection. The Auth 2.0 bearer token allows decoding, verifying, and generating specific credentials, which allows safe transmission of information from one platform to another.

Prior to sending the request to the Postman application (36), the parameters were specified and the Auth 2.0 bearer token of the approved academic project application was added to the app. The data were collected since Twitter was established, i.e., March 2006, until 1 December 2021, using keywords related to the EU FOPL and names of all EU countries. The request-response was received in the form of JavaScript Object Notation (JSON). The related data were extracted to Microsoft Excel 365, including user ID, user description, tweet text, location, date, Re-tweet, Reply-to, tweets-ID, and user-type. The entire original data set is presented in Supplementary File 1. The tweets were composed of eight different languages (English, Spanish, French, Germany, Czech, Croatian, Italian, and Dutch); therefore, the translation process of all tweets into English was performed *via* Google Translate software.

### Data Analysis

After developing the coding manual and training the coders (IA, BA, DWN, NJN, TB, and NM), 10% of the tweets data set was tested based on the clear instructions available to the research team and the definitions provided for each category (attached in Supplementary File 2A). The tweets were manually coded according to the study research questions under the following categories: relevance, announcement, opinion, science, and EU legislation/policy. Tweets were coded as relevant if they were related in any way to food labeling. The opinion category was coded if a person expressed an opinion on any topic related to FOPL systems in the EU. An announcement was coded if the tweet was purely a factual statement or declaration related to FOPL. The science category was considered if the sharing in the tweet was related to or based on science. EU regulation was considered if the tweet was related to FOPL EU policies and presented any comment on EU FOPL regulation. A relevant tweet could fall into one or more categories.

An open discussion was held to resolve differences under the supervision of OV as an external moderator who was not involved in the coding process. The six investigators then proceeded to separately classify the remaining tweets. To assess the inter-rater reliability between six raters and internal consistency of the classification test for tweets, the intraclass correlation coefficient and Cronbach's alpha correlation coefficient were calculated for 10% of the total number of tweets using SPSS 23.

Then, we performed automatic coding of relevant tweets using QSR NVivo. Automatic coding was used to assist the concept development process related to FOPL. After exploring the automatically generated codes, during the content analysis, two researchers (IA and OV) worked together using a combination of inductive and deductive approaches in the generation of codes (37). The codes were manually organized into themes and subthemes whose framework was used for the final analysis.

Dialogues or conversations among two or more people are identified and analyzed separately. Around 20% of the total sample of dialogues was categorized by two coders (IA and DWN), including the participation of an external investigator (OV) to the themes already above mentioned.

### Sentiment Analysis

A sentiment analysis is used to define attitudes and emotions in texts using natural language processing to extract and quantify affective and subjective information. It identified users' opinions and categorized them into three: positive (very positive), neutral (moderately positive and negative), and negative (very negative). The sentiment analysis of relevant tweets was performed using NVivo software (38). Nvivo automatic sentiment analysis, as an automatic sentiment analysis software, is based on expressions of sentiments in the content.

### Network Analysis

A network analysis is an increasingly used methodology, which provides information on the flow and relationship between tweets by specifically connecting the original tweet with the retweet and reply tweet. We used Gephi 9.2 which is an open source software that supports further qualitative investigation by analyzing the connections between the nodes representing the object of interest. In the case of this study, it is a tweet by their edges, which represent the links between them, to exemplify one or more relationships between them, for example, the retweet and reply tweet. The analysis could enable the understanding of how the discussion and interaction were portrayed on Twitter, which involved the use of the main tweet and conversation ID (39). The network analysis included only relevant tweets. A network analysis was also performed for tweets that were part of dialogues and chains of tweets (for example, replies and retweet) and that had a similar conversation ID.

### Geographic Heat Map

Geographic heat maps demonstrate the density of the origin of information. The Twitter data sheet geo location-ID indicates the country and city at the time the tweet was published. We generated the heat map of relevant tweet density from the geo location-ID data using Microsoft Excel's map function (see Supplementary File 2B).

## Results

### Overall Description

The quest produced a total of 4,073 tweets, of which 2,278 (56%) were original tweets, 1,321 were reply tweets (32%), and 474 were quoted tweets (12%). These tweets were sent out by 2,819 different accounts. A total of 229 accounts (4.6%) were owned by institutions or organizations, and 278 (6.8%) to individuals (persons). Entity annotations are defined in Twitter, which are classified as people, organizations, places, products, and others. A total of 3,202 tweets were considered relevant, whereas 871 were irrelevant. A total of 493 were categorized as announcements, 2,624 were classified as opinions, 68 were classified as science, and 165 were deemed to be related to EU policy. The intraclass correlation coefficient was 0.876, which was used to test the inter-rater reliability, considered good reliability (40, 41), while the result was 0.70, which was a generally satisfactory Cronbach's alpha value for measuring the internal consistency of the set of questions used to classify the tweets (42, 43). The intraclass correlation coefficient result for testing the inter-rater reliability of dialogues/conversations was 0.949, which was considered to be an excellent reliability.

**Figure 1:**
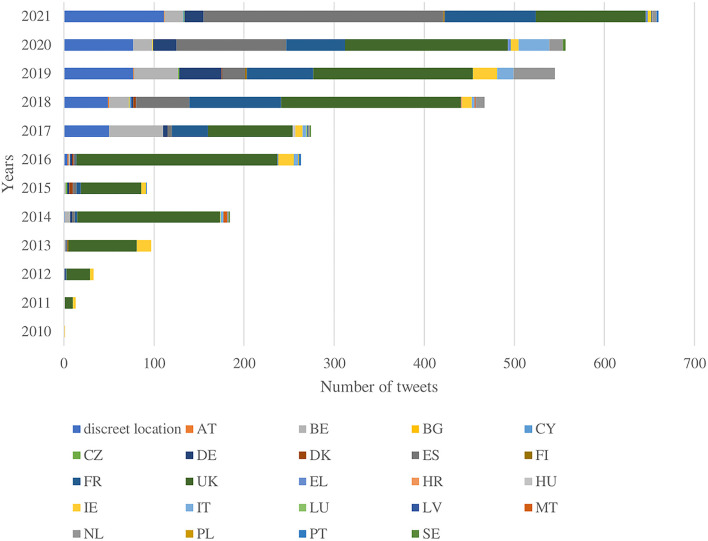
Tweets per country over time. The graph shows the number of tweets per year stacked for each country. Two-letter country codes: Austria (AT), Belgium (BE), Bulgaria (BG), Cyprus (CY), the Czech Republic (CZ), Germany (DE), Denmark (DK), Greece (EL), Spain (ES), Finland (FI), France (FR), Croatia (HR), Hungary (HU), Ireland (IE), Italy (IT), Latvia (LV), Luxembourg (LU), Malta (MT), The Netherlands (NL), Poland (PL), Portugal (PT), Sweden (SE), and the United Kingdom (UK).

On average, EU countries have increased their internet use to participate in social networks over the past decade (creating user profiles, posting messages, etc.). Denmark led in the last 5 years with an average of 81% of the population participating in social networks. In addition to Denmark, the UK, Belgium, Cyprus, Malta, and Sweden have featured in the top five at least three or more times in the last 5 years. Poland, Germany, Bulgaria, France, and Slovenia have had the lowest frequency over the last 5 years, but the lowest rates have always been above 40%. Approximately 63% of individuals from Spain, a country particularly active on this issue, used the internet to participate in social networks in the last 5 years (44). The geographical regions of origin for the accounts were the UK (*n* = 1,434, 44.8%), Spain (*n* = 582, 18.2%), France (*n* = 468, 14.6%), Belgium (*n* = 244, 7.6%), Germany (*n* = 131, 4.1%), Ireland (*n* = 113, 3.5%), the Netherlands (*n* = 97, 3%), and Italy (*n* = 72, 2.2%), 15 countries had <1%, and five countries had zero tweets. Figure 1 shows how the tweeter activity per country use has changed over time. The UK has significant tweeter activity since 2010, with a peak in 2016, followed by Spain and France. The geographical heat map illustrates the intensity of Twitter discussion for the period 2006 and 2021 for EU member states, see Supplementary File 2B. Top 10 most influential users are available in Supplementary File 2C.

### Content Analysis

The major themes are the types of nutritional food labeling, the food industry, healthy vs. unhealthy foods in the context of food labeling, EU regulation, political conflict, and science and education (see Table 1).

**Table 1:** Definition of themes and their subthemes.

**Themes**	**Definitions**	**Subthemes**
Nutritional food labeling types	Tweets discussing any type of FOPL or food labeling.	Nutri-Score and others (Multiple Traffic Lights, warning symbols, allergen, alcohol, environmental front-of-package, origin, etc.)
Healthy food vs. unhealthy food	The theme describes how FOPL relates to different food types.	Unhealthy (junk, fast, UPF, meat), healthy (organic, high nutritional value, vegetable, olive oil, fruits).
Food industry	Tweets on how companies related to food production, manufacturing or selling packaged food are linked to FOPL.	Companies support and accept Nutri-Score, Interests of food industry
EU Regulation	Legislation and regulations with respect to food labeling related to the EU.	Brexit, mandatory/voluntary labeling
Political conflict	Some arguments for or against the FOPL are projected onto countries.	–
Science and education	Scientific facts, publications, appearing on FOPL in tweets.	Evidence about Nutri-Score, scientific articles, value of science, education

Nutri-Score is the number one representative of food labels in the analyzed data set. The dominance of Nutri-Score is also shown by the fact that six of the top 10 influencers are Nutri-Score supporters and only two are explicit opponents. The influencers are representatives of companies as well as individuals, details can be found in Supplementary File 2C. In addition to the Nutri-Score subtheme, the other subthemes covered multiple traffic lights, warning labels, allergens, alcohol, environment, CO_2_ emission label, indication of origin, etc. The usefulness was the most discussed within this theme, there were supportive and rebuttal tweets. The supportive tweets were often general, but also based on personal experience. One example reads: “*Just to add to hunch that much easier to be a coeliac abroad (3rd trip since 2014) than in UK.....GF food readily available at airport!. Feeling very grateful for EU food labeling”* (*80850138*).

Skeptical tweets about efficiency mainly referred to individual responsibility and inconsistencies in food labeling. “*Food labeling will not help or change obesity. Obese people know they are eating more than they need. Some are drinking too much alcohol. Several are consuming too much coke or fanta and such like. People won't read labels and don't understand them. Stop when full”* (*1270909801*).

There was also a general rejection of food labeling: “*How can u be sure of food label as I don't trust them.”* (*1038309122*) or “*Be careful, the subject is not as simple as it seems!* \ *NHow does Nutri-Score, the new food labeling, work: criticisms and virtues of the nutritional traffic light”* (*414143504*).

For Nutri-Score, both supportive and unsupportive tweets are displayed. The support stems from the argument that Nutri-Score has been proven to positively influence consumers' product choice. For example, “*@wirtzbill @Timeo_Danaos #Nutri-Score is on the front of the packaging in addition to the table of nutritional values which remains on the back. It is not the state that decided which information is useful, but the scientific work and consumer demand for N” (831000000000000000)*.

The challenge and debate stemmed from a lack of consistency: can a soft drink be classified the same as a natural juice or a sweetened soft drink, e.g., “*Heated debate about #Nutri-Score. How can you recommend a soft drink with a green B? #fresh #vegetables are the same as processed for a nutritionist. So Mother Nature's packaging is as good as it is from the factory. Can you make it more complicated?*” (*2304682206)*. Others said, “*Diets and overweight have multiplied for years. #Nutri-Score is not a solution, but part of the problem. We should simply eat less refined vegetables, legumes, grains, things that Nutri-Score does not have. And just as it happens, Nutri-Score is often in #plastic packaging” (2200388091)*.

The healthy food subtheme, with the healthy vs. unhealthy food theme mainly showed items from Mediterranean meals, such as olive oil, packaged vegetables, and fruits, or dairy products. The subtheme of unhealthy foods contained junk food, ultra-processed foods, prepackaged and packaged foods, fast foods that are described to be discriminated exclusively by food labeling. This theme has been discussed mainly in the context of Nutri-Score and some think Nutri-Score helps people make healthier choices. For example, “*#Nutri-Score. Choosing the healthiest products will be easier”* (*791731650*). However, even foods considered healthy are easily discriminated, particularly by Nutri-Score. For example, “*The #Nutri-Score doesn't help to the #mediterranean #consumers. This mechanism says that one @CocaCola has a B and one bottle of #OliveOil @AceitesOlivaES has a D*. \ *nSometimes simplifying the system is not the fairest thing to do” (1065958166343660000)*. or “*It seems that Iberian salami and olive oil are less healthy than Nesquik with the #Nutri-Score labeling system. The Mediterranean diet is penalized” (1070000000000000000)*. Traditional foods are not necessarily healthy: “*#Nutri-Score isn't ‘against' GIs. It simply translates the nutritional info already on the back-of-pack*.\*nIt's not because a foodstuff is ‘traditional' that it's nutritionally healthy!*\*n?? More in @beuc Nutri-Score MythBusters …#EUFarm2Fork”* (*2893273911*).

Some argue that Nutri-Score favors industry players to the detriment of healthy products. For example, “*Very well seen: the food industry continues to swallow ultra-processed foods harmful to health while respecting the limits in saturated fat, salt or sugar, therefore with a good rating at #Nutri-Score”(44345678)*.

The theme of the food industry had two main subthemes, one of them was to celebrate the launch of the use of Nutri-Score by food giants. “*Bravo @pepsicofrance for adopting #Nutri-Score and accepting nutritional transparency* \ *n on your products. A victory for consumers and Public Health. When will the same decision be made for Coca, Ferrero, Mars, Unilever, Mondelez and those who still refuse to post it?” (831000000000000000)*.

However, as noted earlier, food giants have less interest in facilitating healthy consumption and may take steps against FOPL: “*Monsanto took the state of Vermont to court to stop food labeling, how did that pan out? But Wholefoods and Monsanto a team scary” (2713126435)*.

Food industry interests can also be seen as the protection of national and local producers. For example, “*Don't get me started, Dee! So many loopholes in labeling. So damaging to the honey and other food industries in Ireland” (2400427131)*. Others also mentioned the price of the new application of food labels, relativizing the public health outcomes: “*The cost of food labeling and packaging in some cases is more than the product itself! What has it achieved? Very little!” (2400427131)*.

Food industry is often considered clearly responsible for the consequences of unhealthy food consumption. For example, “*Food packaging laws are a farce. Instead of truthful packaging, we get packets of lies—from a food industry raking in billions at the expense of the nation's health. Today, on World Diabetes Day, it is time we pledged to do something about it” (14190551)*. Some believe that even if regulation does take place, it may be because they have been able to reach an agreement with policymakers, thus skepticism of policymakers is also expressed, e.g., “*Well, Nutri-Score is crap designed for the benefit of food companies, not the consumer. That's the important thing, not political wars (everyone has supported it sooner or later)” (713000000000000000)*. On the other hand, others believe that the food industry is blocking the successful implementation of Nutri-Score: “*Sensitive to the lobbying of a few companies, 7 European countries are still opposed to the European #Nutri-Score, a precious tool for strengthening the power and the faculty of discernment in terms of consumer health. Europe must not give in to the pressure!” (167328296)*. The shared interests of politics and the food industry also appeared in tweets, e.g*., “Specific national mandatory rules on origin labelling is a challenge both to the single market and to the burdens of food business operators!” (516260680). “Vytenis Andriukaitis, European Commissioner for Health and Food Safety”* (*516260680)*.

Tweets related to the theme of EU legislation showed mainly events, conferences, and announcements, factually. For example, “*#ProNutri-Score 2,286 signatures to go to reach 50,000! Let's do it over the weekend?*\*nYou can sign the European Citizens Initiative calling for #Nutri-Score to become mandatory across the #EU here:* http://www.pronutriscore.eu/”* (2893273911)*.

Those who were evaluative tended to be in favor of EU legislation because of its impact on the internal market or its positive influence on individuals. For example, “*I welcome @SKyriakidesEU commitment to include food labeling as part of the #FarmtoForkStrategy That's a clear ask of EU citizens and we are ready to have an EU wide front packaging information to help consumers to make the right choice” (135430876)*. The harmonization of FOPL and a single-market approach are welcomed by most tweeters even if the symbolic Nutri-Score was opposed, e.g., “*We all know what we do not like (Nutri-Score !) but will we agree on a European approach?#evolvednutritionlabeleu*” (*32307080*) or “*Food labeling benefits from EU harmonization. Confirmed during the Belgian Food industry association @fevia_be's event” (455775907)*.

The mandatory or voluntary nature of the regulation is also addressed in the tweets, with a significant number of contributors in favor of mandatory. For example, “*@fergril In Europe there were several front labels and none of them were warnings. The fact of achieving a harmonization with the #Nutri-Score between the different countries, I hope it will be the push to change the regulations and achieve the obligation” (273084024)*. Others disagree, e.g., “*@pcanfin @SKyriakidesEU Harmonised labeling across Europe is a must to avoid confusion. The right choice?... not sure... On nutrition labeling is really about nudging the right consuming behavior and educating. And on sustainability... there should be” (32307080)*.

The Brexit subtheme was raised in several tweets, which had a significant impact on FOPL regulation in the UK. For example, “*Post #Brexit the govt would relax food source labeling to “support” public. There would be zero chance to identify hormone produced and select safe alternatives from smkt shelf. The plus side: next generation could be taller than #EU cousins: all above 2m. EU safeguards???? (715760036).”*

In a broader sense, the tweets depicted a political conflict with different reactions from individuals, political, and governmental actors, in which agribusiness interests and the public health interest collide. Most tweets contained pessimistic, questioning, and negative comments. For example, “*Nutri-Score: It's good when you can control the algorithm, says @JGurkmann @vzbv. However: Nutri-Score is a model and a brand of the French state. The right to have a say is questionable. #kostbar” (1671248730)*. Politicians take action to protect their interests as “*Taking advantage of the last month of its European presidency, Germany intends to change its agenda to push Europe to adopt the #Nutri-Score in agreement with France to attack the #MadeInItaly” (348979316)*. The tension seems to be between pro Nutri-Score countries and the Mediterranean countries, e.g., “*The #Nutri-Score model is a deceptive system that damages #madeinItaly. We work on a fair and clear system that protects consumers and producers. I talked about it this morning at the “Made in Italy in Europe” event, here in the European Parliament with the #Aepi Confederation” (1368850908). “The NutrInform Battery nutritional label on the front of the packaging label proposed by Italy is also considered misleading “Italian government notified its ‘#NutrInformBattery' label to @Food_EU* https://t.co/e96jmnx5as *to counter momentum for #Nutri-Score*.\*nYet scheme is confusing and counter-intuitive: highly charged batteries may wrongly interpreted as a goal to achieve! Not helpful. #FarmToFork*” (2893273911). The importance of the protection of local products seems very influential, e.g., “*@agarzon Let's see if what Riolobos or Villalobos was referring to or whatever it is called, is that he advocated the creation of a similar labeling system, BUT SPANISH, NOT FRENCH, that would not lower the quality of Spanish products, as it does Nutrisco” (290000000000000000)*.

Political differences have been discovered not only between countries, but also specifically targeted against certain politicians, e.g., “*Alberto, you're probably the guy's asshole the current government, and it is not easy eh. Nutri-Score system is rubbish and here we are going to implement because it is customary to eat the ass to the European Union if somewhat detrimental to the country and citizens.” (165884355) or “Food Minister Klöckner's decision in favor of #Nutri-Score food labeling is correct, but it is only a small step—and it comes very late” (488614084)*.

A relatively small number of tweets under the science theme drew attention to the fundamentals of Nutri-Score science, some referred to scientific articles and talked about the value of science, e.g., “*It is important to highlight that the #Nutri-Score is a system that was born from the academic field, and regardless of any type of conflict of interest with the food industry. Science should be the only argument to select the labeling model!” (273084024)* or “*Research reveals that #Nutri-Score enable people to make better nutritional decisions. We're conscious manufacturers have a role to play, that's why we're embracing this #Transparent and consumer-friendly labeling system to promote a #healthier #Diet” (1190000000000000000)*.

Another subtheme covered tweets related to the health education of population, urging actions toward this direction. “*#Prevention and #health education work! According to a simulation by the Epidemiology Research Team, #Nutri-Score would prevent nearly 8,732 deaths per year. It is important that manufacturers commit to its deployment!”* (*2279236686)*. Others underlined the need for education after the implementation of FOPL, e.g., “*?? The implementation of #Nutri-Score will require an educational campaign for professionals and the population? Essential point: your diet should be based on fresh and healthy food” (273084024)*. Some tweets described how such education should be carried out: “# Taboo words in # nutrition education: “*Communicate in a contemporary way! Is there right and wrong diet? And what is actually healthy? Why certain terms are obsolete when we talk about #eating and #drinking?? …# Nutritional communication” (22252511)*.

For greater specificity, the dialogues were identified and analyzed to see which were the most common themes. The number of dialogues/conversations identified was 327. The most frequent tweet theme was label type (97.55%), followed by the food industry (26.91%), bad food (18.96%), EU regulation (14.06%) science (11%), political conflict (10.09%), and healthy food (9.78%).

For a better understanding, organizations and persons were also assessed using major themes, their analytical table is available in Supplementary File 2D.

### Sentiment Analysis

We identified the feelings associated with the major themes. Feelings were classified according to four categories: very negative, moderately negative, moderately positive, and positive with the number of counts (see Table 2). Moderately negative and moderately positive were considered as neutral or mixed feelings. As a whole, the text had rather negative sentiment. Food industry tweets had the most positive sentiment, while political conflicts received the most negative sentiment. The Sentiment analysis of the discussion of tweets from organizations and individuals is available in Supplementary File 2E.

**Table 2:** Sentiment analysis of themes.

	**Very negative**	**Moderately negative**	**Moderately positive**	**Very positive**
Label types	18.33%	37.12%	29.17%	15.38%
Healthy vs. unhealthy foods	23.63%	32.63%	28.05%	15.7%
Food industry	17.02%	26.95%	31.06%	24.98%
EU regulation	22.92%	36.87%	20.43%	19.78%
Political conflict	26.11%	34.28%	20.41%	19.2%
Science	16.6%	39.74%	26.66%	17%

### Network Analysis

**Figure 2:**
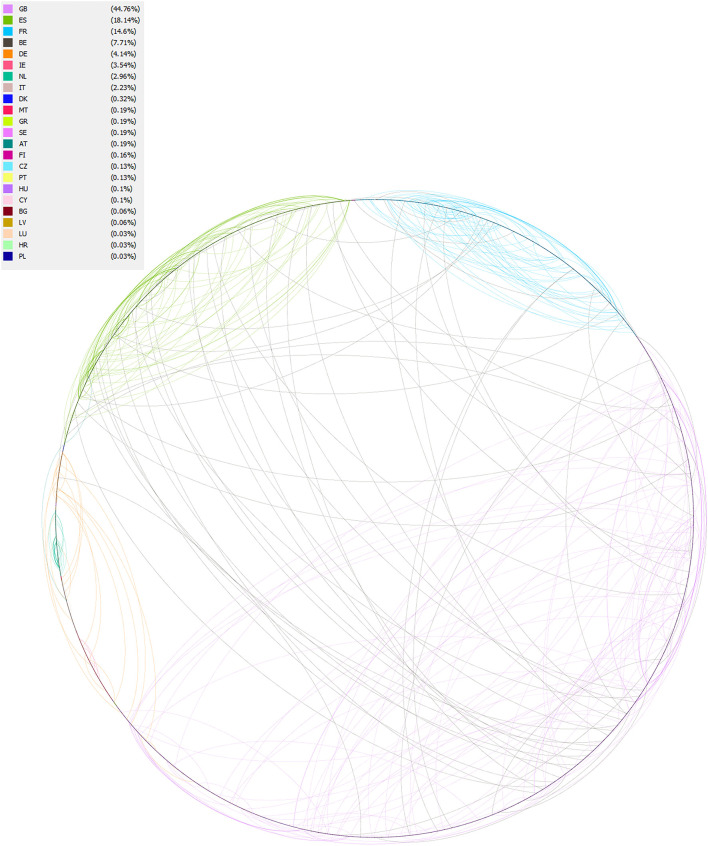
Network analysis. The circumference of the circle shows the total amount of tweet data. The circumference is divided into segments, each color corresponds to a country and each dot is associated with their conversation ID. The segments are connected by chords that illustrate the relationship within/between countries. Each tweet relationship is symbolized by a separate chord.

The chord chart (Figure 2) shows the communication link within and between countries. The network analysis had only 3,138 nodes and 626 edges. The figure indicates that communication between countries was limited and tweets were mainly limited to within countries. Most connections were observed in France.

**Figure 3:**
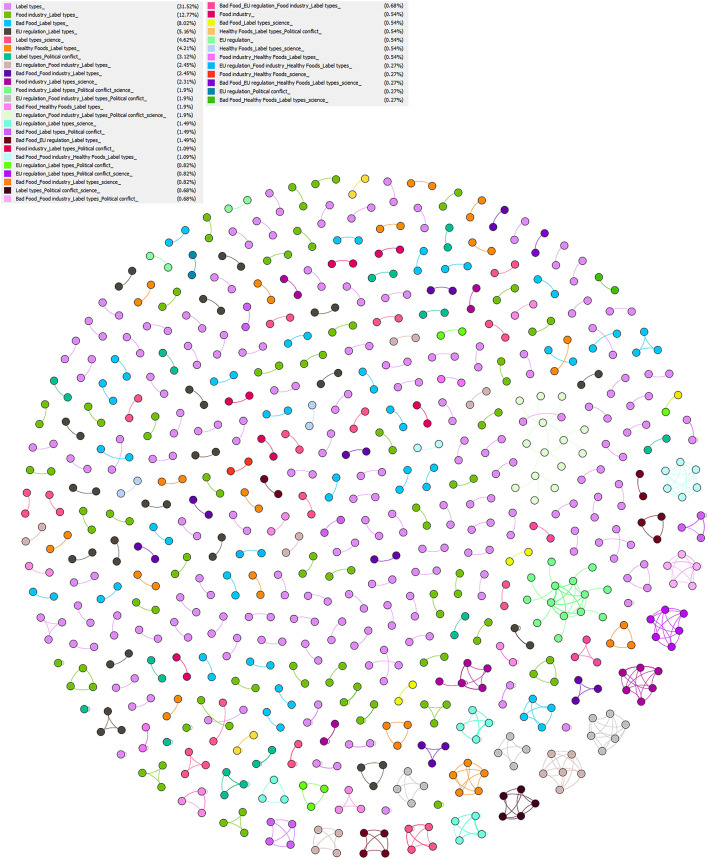
Network analysis of dialogues. The Fruchterman–Reingold layout algorithm shows tweets belonging to dialogues and having similar conversation IDs. The dialogues were represented by colors, each corresponds to the different themes in which the dialogues were coded. The nodes (tweets) are connected by lines, representing their connections.

The result of the network analysis of dialogues/conversations included 736 nodes and 581 edges. The nodes represented the tweets and the edges represented the connections of the tweets that were part of dialogues. Each dialogue was divided into several coded themes, as presented in Figure 3.

## Discussion

To the best of our knowledge, this is the first manuscript to evaluate the public discourse on Twitter about FOPL in the EU. Our analysis showed low Twitter activity, despite the high public health importance of the topic. The topic on Nutri-Score dominates the public discourse on Twitter, which is in line with the observations by Mazzu et al. (17) compared to, e.g., multiple traffic lights, warning labels, and reference intake, and to more general keywords (FOP, FOPL, and front-of-pack). Indeed, our results suggest that it is often not the FOPL system in general, but largely the validity of the Nutri-Score that is at the heart of the debate.

One of the biggest dilemmas for tweets was whether it is worth introducing a very simple color label with a clear message and no nutrition information (9). A better-rated FOPL can also be applied to ultra-processed foods with poor nutritional value as sugar-free soft drinks, as repeatedly published in tweets. FOPL that are based on an algorithm or score, such as Nutri-Score, multiple traffic lights, and health star rating, do not necessarily distinguish between nutritionally recommended and less recommended foods, such as whole grain and refined grain foods (45). To resolve the dilemma between using nutrition labels and recommending traditionally healthy foods, for example, Israel has chosen an interpretive FOPL system using two colors to indicate negative or warning (red) and positive (green) labels. While the warning FOPL is mandatory, the positive FOPL is voluntary. The absence of a warning label does not mean that a product is recommended for consumption (45, 46).

The food at the heart of the Nutri-Score debate is the symbolic food of the Mediterranean diet, olive oil. Many players in the olive oil sector suggest that the “Yellow C” label assigned to any olive oil by Nutri-Score does not adequately reflect the documented health benefits of extra virgin olive oil, implying that the label misleads the consumer. They argue that extra virgin olive oil should be assigned to the best healthy food category, “Green A” (47). The European Commission launched a public consultation on proposed changes to food labeing to help consumers make healthier and more sustainable food choices and fight food waste. Although stakeholders have until March 7, 2022 to submit their views, 214 valid responses have been received so far, many from olive oil producing countries (48), indicating lobby activity.

Front-of-pack nutrition labeling systems have a significant impact on the marketing strategies of large agribusinesses. For example, in June 2021, Nestlé—often associated with healthy foods—became embroiled in controversy when an internal company document was made public showing that 60% of Nestlé's core food product portfolio was unhealthy (49). According to those who tweeted, the industry's strategy is simple; they change the composition of foods to rank them higher, but making this change does not necessarily lead to better nutritional value (50).

The general topic was perceived negatively by Twitter users but positive sentiments toward the food industry were observed. Positive sentiments toward food industry were likely partly due to the Twitter activity of these companies, partly due to tweets acknowledging the adoption of Nutri-Score by several food companies.

The food industry's lobbying strength is well-documented around the world, so the emergence of this theme in the Twitter discourse is not surprising. During the adoption of nutritional warning labels in Colombia, a research was conducted to detect and track the use of political practices by the food industry. According to their results based on Twitter posts, document analysis and interviews, food industry players have lobbied Congress and pushed their own agenda in the media to try to prevent the adoption of warning labels in Colombia (51, 52). Similarly, an Australian study found that the ultra-processed food industry has actively used Twitter to influence food and health policy debates. Seven broad strategies were identified in tweets: combining public health narratives; opposing regulation; promoting voluntary, co-regulation or self-regulation; engaging the political process and decision-makers; linking the regulatory environment to the need for continued profitability; influencing public perceptions and value judgments; and using ignorance claims to distort policy narratives (53).

The EU has determined that increased consumer knowledge will lead to more accurate, educated, and easier to comprehend food decisions (54). Although a number of tweets presented the EU's overall objectives for FOPL, we found virtually no communication between EU actors and other stakeholders. Furthermore, we found no evidence of actual EU educational activities on FOPL on Twitter. The lack of confidence in EU regulation is concerning although it is by no means limited to this area. For example, for people to follow the directives given by the government, trust is required between the two parties, as demonstrated during the COVID-19 pandemic (55). The FOPL-related EU regulation was a special case of the Brexit and its consequences in the UK. The UK wide voluntary scheme, which was in line with the EU FIC introduced in 2013, combined color coding and percentage reference intakes. The labeling has been applied by two-thirds of the packaged food and beverage market. Following Brexit, the UK government announced that it intends to apply FOPL nutrition labeling after reviewing its effectiveness and exploring alternative schemes (56).

Twitter is known as being the word-of-mouth (WOM) network for digital discussions. Because there are so many users and so many tweets sent each day, social media networks like Twitter have been proven to be more effective in disseminating information and engaging in extensive conversations on issues of public concern than formal communication and marketing. The model that Twitter WOM uses called “organic” communication, happens without direct marketing prompts, influence, or measurement. This can be explained in the scenario when an influencer and a follower are enmeshed organically, and they engage by replying to a tweet or retweeting. With a high number of retweets and a large number of followers on social media, the user becomes a social influencer and therefore their tweets go viral. When these tweets go viral, it has been proven to boost the visibility of campaigns and ongoing conversations. From this evidence, Twitter has proven to be an effective platform for WOM campaigns, which could be used effectively for FOPL's social network discourse (57, 58).

The WOM in social network debates around FOPL discussions attracts many people as consumers play a crucial role in communication and information transfer, which is known as the “social customer journey.” Customer journeys play a key role in how people share ideas, follow some issues, or fight some problems on social networks. In relation to FOPL, social networking discussions on Twitter are heavily influenced by the customer experience, that is, the customer journey. Consumer discourse and FOPL regulations are closely linked (59). In Europe, as countries makes their own preferences, there is no one-size-fits-all solution, and the shortcomings and gaps of FOPL have delayed its implementation, (60, 61).

Although the EU intends to accept a harmonized FOPL before the end of 2022 to tackle obesity and other non-communicable diseases (NCDs), the ideal approach is highly debated among member states (62). Summary indicator-type Nutri-Score is the most popular and has been debated at high political levels, especially in Italy and other Mediterranean countries, as mentioned above (63). Although there were little or no further political conflicts between countries in the tweet messages, there are additional political conflicts over the harmonized FOPL. Seven EU member states—Italy, Cyprus, Greece, Hungary, Latvia, Romania, and the Czech Republic—have indicated that if a new harmonized nutrition labeling system with FOPL labeling is adopted, it should be in line with the text of the FIC regulation, i.e., it should provide factual information on the individual nutrients in a product and, therefore, exclude any system that would give an overall assessment of food, such as the Nutri-Score. Other member states, e.g., France, Belgium, Spain, Germany, the Netherlands, and Luxembourg, support the exemption of products of protected origin and products containing a single ingredient (64). The theme of political conflicts encompasses many negative emotions, as evidenced by the fact that it has proven to be the most negative in the sentiment analysis.

Although scientific results are often published on Twitter, this is not the case for our data. There is a broad consensus that health claims need to be meaningful to consumers and be scientifically substantiated and reliable, but should not use overly sophisticated scientific wording. According to scientists, consumers' knowledge of existing health claims needs to be improved (65). Future research should focus on the problems across FOPL systems and on nutrition label education that improves understanding and application, which may improve the impact of this information on dietary health choices (66).

In summary, a harmonized FOPL system will be introduced in all EU member states according to an EU proposal from 2020 (10, 19). Such a system is considered as an important tool to tackle obesity and NCDs as nutrition information should not be based solely on the less efficient ingredient list of prepackaged foods. Whatever FOPL system is implemented (67), it is important to ensure that it is an integral part of the response to obesity and disease is important. Our results made it clear that discussions about FOPL on Twitter are limited to a narrow circle; therefore, consumers' education on FOPL should be emphasized, making people aware of existing and upcoming FOPL systems (68). Educational programs should empower consumers to understand what a healthy diet is and how FOPLs are relevant to national nutritional guidelines.

### Limitations

The text limit on Twitter is quite short (a maximum of 280 characters), which limits the expression of viewpoints and provides background information that inevitably has implications for content analysis. The tweets were posted in different languages; this barrier may have contributed to limited connections between countries. Additionally, machine translations might distort the original meaning of some tweets. Regarding sentiment analysis, it is crucial to note that Nvivo does not classify content based on sentiment. Sentiment is considered by isolated words without taking context into account. Like most text analysis tools, Nvivo does not detect sarcasm, double negatives, slang, dialect variants, idioms, ambiguity, etc. Another limitation was the network analysis, which was intended to represent the country by showing each user ID and the required coordinate data. The data obtained from the full archive of Twitter API Postman research with the long search period from March 2006 to early December 2021 had many irregularities, especially most of the tweets did not have coordinates to indicate where the tweet was posted from. Our access to user characteristics, e.g., number of followers, was limited, so the characterization of accounts was not undertaken, which might have prohibited us from filtering out fake accounts. Additional limitation is that, although the overall lack of trust in the internet and social media information sources is significant across EU member states, trust surveys are context-dependent and reveal significant variations (69). Due to the lack of data on the impact of distrust on these sources in EU policy discussions, this could not be incorporated into the analysis.

## Data Availability Statement

The original contributions presented in the study are included in the article/Supplementary Material, further inquiries can be directed to the corresponding author/s.

## Ethics Statement

Written informed consent was not obtained from the individual(s) for the publication of any potentially identifiable images or data included in this article.

## Author Contributions

AS, NM, BS, DW, and OV contributed to conceptualization. AS, NM, BS, DW, NN, and BT contributed to data curation. AS, NM, BS, DW, NN, and OV contributed to formal analysis. OV contributed to supervision and funding acquisition. AS, NM, BS, NN, and OV contributed to methodology. AS, NM, BS, DW, BT, and OV wrote the original draft, contributed to writing, reviewing, and editing the manuscript. All authors have read and agreed to the published version of the manuscript.

## Funding

AS and DW were supported by the Stipendium Hungaricum Scholarship. OV received fellowship from the Hungarian Academy of Sciences (Premium Postdoctoral Research Program) Grant No. 3134/2019/KP.

## Conflict of Interest

The authors declare that the research was conducted in the absence of any commercial or financial relationships that could be construed as a potential conflict of interest.

## Publisher's Note

All claims expressed in this article are solely those of the authors and do not necessarily represent those of their affiliated organizations, or those of the publisher, the editors and the reviewers. Any product that may be evaluated in this article, or claim that may be made by its manufacturer, is not guaranteed or endorsed by the publisher.
