# Supplementary material for: Analysis of Content, Social Networks, and Sentiment of Front-of-Pack Nutrition Labeling in the European Union on Twitter
Source: Front Nutr. 2022 Apr 25;9:846730. doi: 10.3389/fnut.2022.846730 (PMC9083270; doi:10.3389/fnut.2022.846730)
Supplement: Supplementary file 2 [file Table_2.DOCX]

**Suppl. 2A. Categories and definitions manual coding guidance**

| **Category** | Definition |
| --- | --- |
| Relevance | If tweets were in any way related to food labelling (Nutri-score, color-coded nutrient schemes, etc.), code 1. If the tweet is irrelevant code 0. A relevant tweet continued to be coded and could fall into one or more sub-categories. |
| **Subcategory** |  |
| Opinion | If a person expressed an opinion on any topic related to food labelling systems in the EU; any sentiment expression, view or judgement not based on knowledge or scientific fact code 1. If the tweet is irrelevant to opinion, code 0. |
| Announcement | If the tweet was purely a factual statement or declaration related to food labelling system, code 1. If the tweet is irrelevant code 0 |
| Science | If the sharing in the tweet was related to or based on evidence-based science, code 1. If the tweet is irrelevant code 0 |
| EU regulations | If the tweet was related to food labelling EU policies and presented any comment on EU food labelling regulations or any legal act, code 1. If the tweet is irrelevant code 0 |

Description of Suppl.2A. The list of categories used for guidance to explaining the criteria and definitions that were applied for manual coding. One main category is Relevance followed by 4 categories (Opinion, Announcement, Science, EU Regulations).

**Suppl. 2B. Heatmap of Twitter activity in the EU member states between 2006 and 2021**


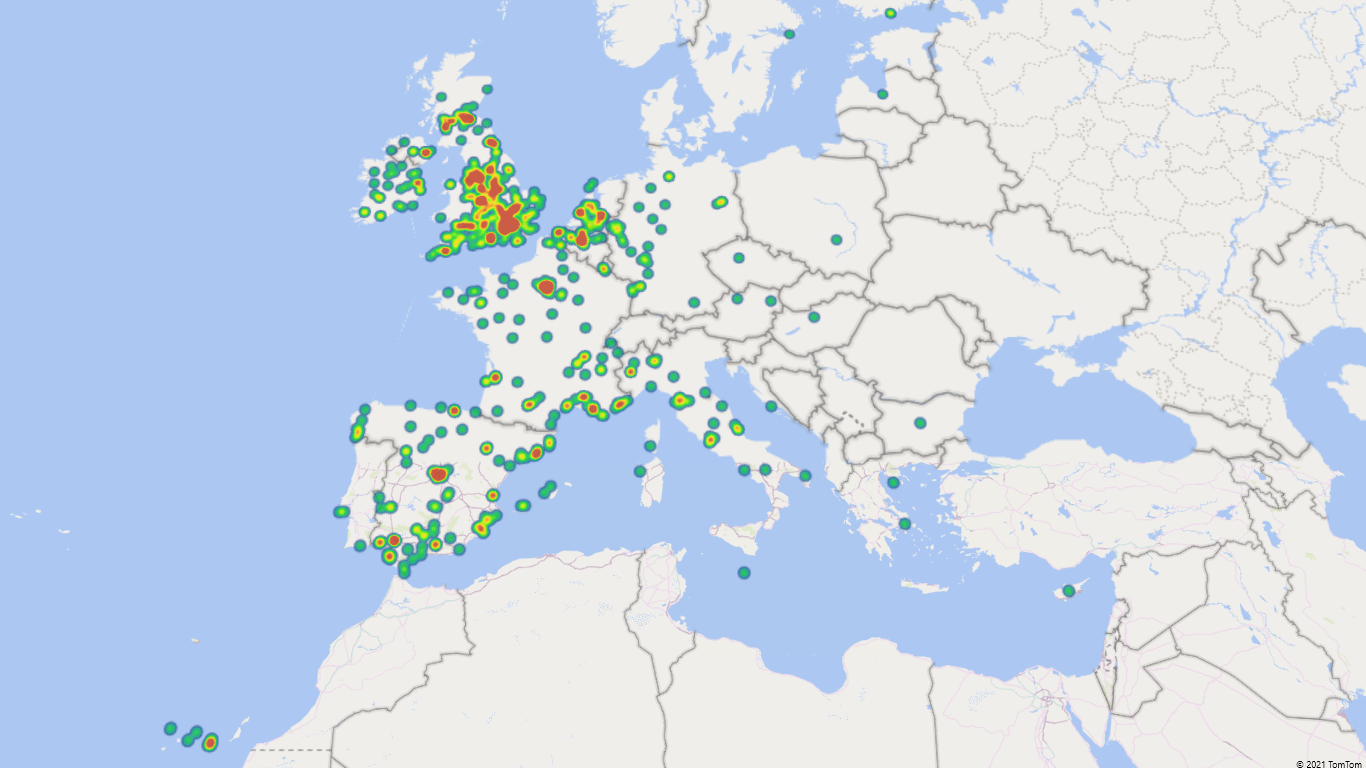


Description of Suppl.2B. Heatmap Twitter activity among EU member states period 2006 to 2021. Heatmap showed the density of tweets based on user location when tweets were posted. Red, yellow, and green in the order displayed high to low density. The Highest density portrayed in UK.

**Suppl. 2C. Top 10 most influential users**

Description of Suppl.2C. Top 10 most influential users based on the total number of followers of Re-tweet, Reply to tweet and quoted tweet. The most influential users in EU dominated from 4 countries (Spain, France, United Kingdom, Germany).

**Suppl. 2D. Percentage of themes of Tweets by organizations and persons**

|  | organization | person |
| --- | --- | --- |
| Bad food | 52,45% | 47,55% |
| EU regulation | 74,14% | 25,86% |
| Food industry | 52,78% | 47,22% |
| Healthy food | 51,05% | 48,95% |
| Label types | 59,72% | 40,28% |
| Political conflicts | 65,41% | 34,59% |
| Science | 41,76% | 58,24% |

The Supple 2D shows the tweet discussion from organizations and persons according to 7 themes. Users labelled as persons were more likely to focus on science and food quality while organizations were discussing EU regulations and political conflicts.

**Suppl. 2E. Sentiment analysis Tweets by organizations and persons**

|  | *Very negative* | *Moderately negative* | *Moderately positive* | *Very positive* |
| --- | --- | --- | --- | --- |
| *Organization* | *23,22%* | *37,57%* | *24,52%* | *14,69%* |
| *Person* | *30,32%* | *28,35%* | *23,97%* | *17,35%* |

The Suppl. 2E presents the sentiment analysis for tweets from two categories (organization and person) by four type of sentiments. Sentiment analysis showed that individuals had more negative and more positive sentiments than the organizations.
